# Does the Mortality of Individuals with Severe Disabilities Contribute to the Persistent East–West Mortality Gap Among German Men?

**DOI:** 10.1007/s10680-022-09609-4

**Published:** 2022-03-01

**Authors:** Olga Grigoriev, Gabriele Doblhammer

**Affiliations:** 1 Max Planck Institute for Demographic Research, Rostock, Germany; 2 Institute for Sociology and Demography, University of Rostock, Rostock, Germany; 3 German Center for Neurodegenerative Disease, Bonn, Germany

**Keywords:** Mortality, Disability pension, Regional differences, Germany

## Abstract

After three decades since reunification male life expectancy in East Germany still lags behind that of West Germany. Unlike most of the prior studies focusing on the role of socioeconomic factors, this study aims at assessing the contribution of the population with severe disabilities to the persistent East–West male mortality gap. Our analysis is mainly based on the German Pension Fund data. It is restricted to men aged 30–59 receiving disability pension (DP). We estimate mortality indicators and compare trends among populations with or without DP. We use decomposition method to quantify the effects of changes in mortality and compositional changed in the prevalence of receiving DP on the East–West mortality difference. The analysis covers the period 1995–2013. The German Socioeconomic Panel data and Cox proportional hazard models are used to evaluate the regional differences in the risk of receiving DP. Our results suggest that both the higher prevalence of receiving DP in the East and the higher mortality level among men not receiving DP in the East explain the East–West gap. The mortality difference among those receiving DP is negligible and does not contribute much to it. The observed higher prevalence in receiving DP in the East is very likely to reflect the reality as we found no regional differences in the risk of transitioning to receiving DP. The disadvantageous position of the East can be explained by the post-reunification crisis which particularly hit young men in the 1990s, selective migration from East to West after reunification, and the higher proportion of the healthier foreign population living in the West.

**Supplementary Information:**

The online version contains supplementary material available at 10.1007/s10680-022-09609-4.

## Introduction

**Fig. 1:**
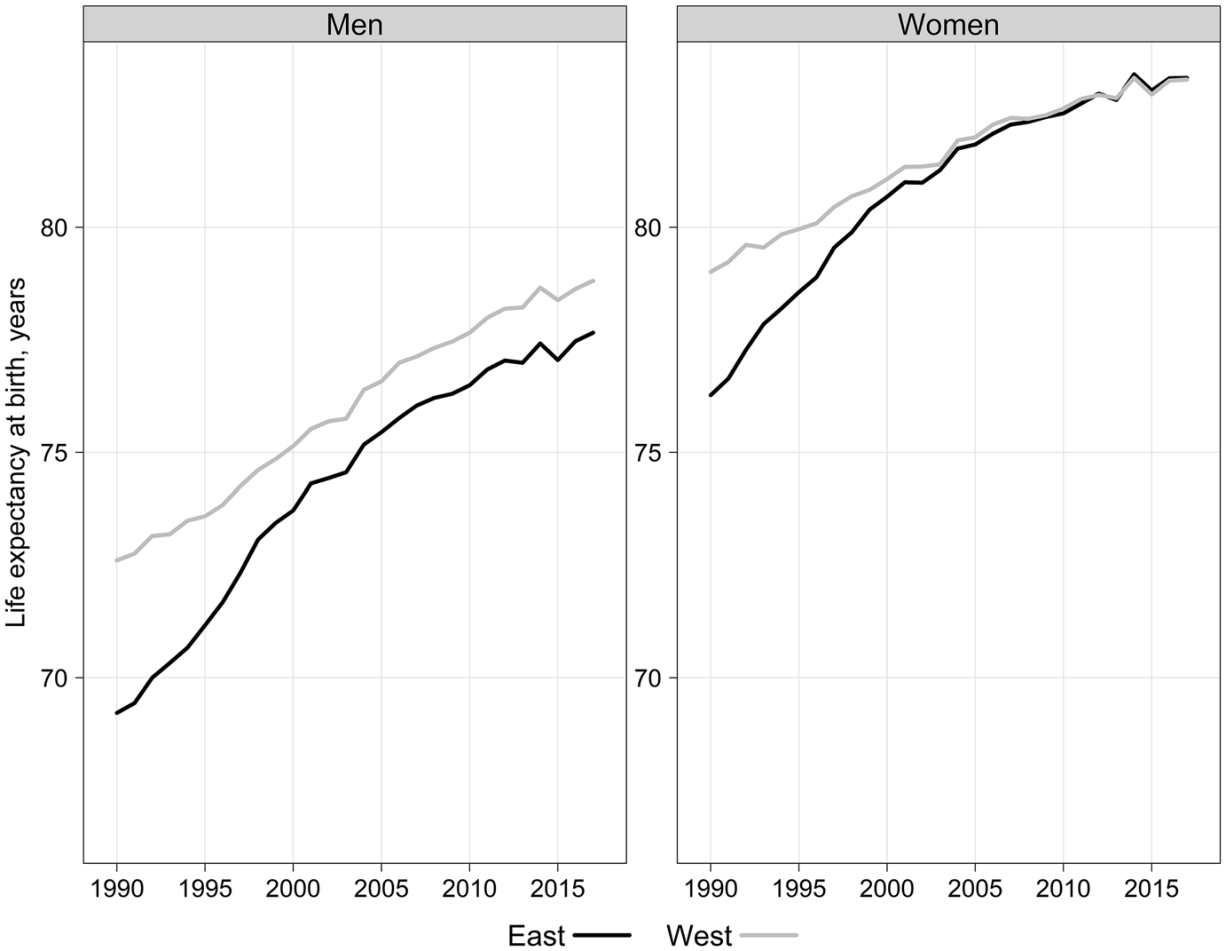
Life expectancy at birth in East and West Germany, both sexes, 1990–2017 Source: Human Mortality Database (HMD)

In the last two decades, the mortality trends in East and West Germany have been developing differently for women and men. Female life expectancy at birth in the East had risen to Western levels by the early 2000s, but the East–West gap in male life expectancy has yet to close (Fig. 1).

The East–West difference in male life expectancy was 3.4 years in 1990. It declined to 1.1 years by 2007 and has since remained almost constant. This East–West mortality gap has mainly been driven by men of working ages (Scholz et al., 2010; Scholz, 2011). As Grigoriev and Pechholdova (2017) show in their work, alone in 2013 about two-thirds of the difference was due to mortality among men aged 30–59 years.

### Previous Research on the East–West Mortality Difference

Previous research focusing on the contribution of causes of death to mortality gap between two parts of Germany among working-age men has suggested a decreasing role of external causes of death and an increasing role of non-communicable diseases (especially heart diseases and cancers). For example, the results of Grigoriev and Pechholdova (2017) show that external causes of death were important among adult men in the post-reunification period: In 2000, they accounted for about 29% of total difference (estimated from results in Grigoriev and Pechholdova, 2017). However, over time, they have become less important, falling to 17%. The contribution of external causes was especially high among the ages 15–29 years. In more recent years, the persisting gap in male mortality has been driven by higher mortality from heart diseases and cancers in East Germany (Grigoriev and Pechholdova, 2017).

Previous studies have suggested a rapid convergence of mortality rates between East and West Germany after reunification, associated with improvements in the economic situation in East Germany (increase in per capita income, pension reform and introduction of a 1:1 exchange rate between two regions) (Diehl, 2008). At the individual level, the differences mainly reflect variations in the characteristics of the respective populations, including age, employment status, health insurance status, migration background or nationality (Scholz, 2010; Scholz et al., 2011). The survival advantage observed among West German men of working ages almost disappeared when individual characteristics such as income, education and employment status were controlled for (Grigoriev et al., 2019). It has been argued that the adoption of Western health behavior and improvements in health care in the East after reunification (Kiebele, 2012; Diehl, 2008) contributed to the decline in the health inequalities between the two regions (Kühn et al., 2019).

While German reunification had some positive effects on economic conditions in the East, it also had some negative effects on the region. Following reunification, levels of unemployment (Kruger & Pischke, 1995) and social stress (Mielck et al., 2000) increased in the East. The previous literature has suggested that younger working cohorts suffered the most from the changes in labor market situation (Geiβler, 1996; Roelfs et al., 2011; Simonson, 2015 in Kühn et al., 2019), while older men from the East benefited from changes in the pension system and improvements in health care (Simonson, 2015 in Kühn et al., 2019). Moreover, large numbers of well-educated young people left the East to pursue better job opportunities in the West (Berlin Institute for Population & Development, 2015).

It should be noted that other factors likely contributed to this East–West gap, including selective migration, lifestyle choices, and the prevalence of various diseases and health conditions (Kibele et al., 2015). However, while East–West and West–East migration may be able to explain the divergence in life expectancy between the two German regions, it cannot explain the post-reunification convergence (Diehl, 2008). The migration effect was probably balanced out by other factors, including the positive effects of medical progress, lifestyle changes, and reductions in cardiovascular risk factors (Luy, 2004).

However, whether and, if so, how the adoption of Western health behavior and improvements in health care in the East may have contributed to the remaining gap in mortality between Eastern and Western German men remains unclear. Do people in the East have worse overall health, or is the East–West mortality gap attributable to a smaller share of the population having severe health problems in the West than in the East? Additional research is needed to evaluate how the share of people with severe health limitations or disabilities has affected the mortality differences between the two regions.

### Previous Research on Disability Risks

It is generally acknowledged that disabled individuals face a much higher risk of mortality than the general population (Karlsson et al., 2007; Majer et al., 2011; Pongiglione et al., 2016). Björkenstam and colleagues (2014), for instance, analyzed data from the population-based cohort study with a 14-year follow-up of almost 5 million people aged 16–64 for Sweden and found that mortality was higher among people who were receiving disability pension benefits than among those who were not, regardless of sex, age, several socioeconomic factors, and previous use of health care measured as days of inpatient care.

The observation that disabled retirees face a higher mortality risk than non-disabled retirees was also confirmed by Polvinen and colleagues (2015). They used data for people aged 30–64 in Finland to evaluate socioeconomic inequalities among disabled retirees, while taking the effect of the disease that led to the disability into account. They found that mental health problems and diseases of the cardiovascular system were the main causes of death in this group, and that socioeconomic inequalities in mortality were most pronounced among those who retired due to disability.

In Germany, qualifying for a disability pension is the main path to early retirement (Hanel, 2010). Evidence for Germany that disabled pensioners had much higher mortality than non-disabled pensioners who retired earlier than the official retirement age has been reported by Brockmann and colleagues (2009). Based on data from one of the German health insurance funds (Gmüder Ersatzkasse) for 1990–2004, the authors acknowledged a dual impact of early retirement: on the one hand, those who retired early due to disability had higher mortality risks; while on the other, those who retired early but remained healthy did not have shorter lives than those who retired later. Mortality risk is confirmed to change with age: male pensioners collecting disability benefits who left the labor market between the ages of 51 and 55 had much higher mortality risks than those who retired at ages 61–65.

Higher mortality among people receiving DP in Germany was also confirmed for the younger ages: Men aged 30–59 receiving DP had nine times higher mortality that it was for the actively insured (healthy) men of the same ages covered in the German Pension Fund (Deutsche Rentenversicherung Bund, DRV) data (Grigoriev et al., 2019).

When a relatively large share of a country’s population is receiving DP, the country may experience a labor shortage, and its economy may suffer. For this reason, much of the domestic literature on this topic has focused on the socioeconomic differences among people receiving DP, and the effects of these differences on the living conditions of individuals (Riphahn, 1999; Hagen et al., 2010).

Riphahn (1999) when analyzed the incentive effects of DP among West German men in 1984–1991 concluded that the impact of DP on retirement due to disability was small, which might be attributable to the availability of alternative benefits (e.g., unemployment benefits). An individual’s age, health, and wages before receiving a DP were found to have much stronger effects on the likelihood of transitioning to disability retirement. Similar results were reported by Hanel (2010), who evaluated the impact of the 2001 pension reform on retirement behavior and found that concerns about retirement benefits had only a small effect on the decision to apply for DP, and that the individual’s health status and expected wages were the two main forces driving this decision.

Previous research has suggested that having lower qualifications increases the risk of collecting DP, especially when the individual’s diagnosis is considered (Hagen et al., 2010). The authors utilized the DRV data for 2004–2006 and confirmed a large role of all of the musculoskeletal disorders and diseases of the circulatory system in the risk of receiving DP, particularly among East German men. The growing incidence in cardiovascular and musculoskeletal diseases observed among East German men is suggested to be explained by the disproportionate numbers of these men working in manual jobs with stressful environmental conditions (Hagen et al., 2010). Previous research has also suggested the high risk of disability due to chronic health problems associated with mental disorders and somatic illnesses in Germany, in general and among people aged 55–60 and men in the East, in particular (Hagen & Himmeireicher, 2014).

The official statistics of the German Pension Fund shows that the non-communicable diseases are the main causes for receiving pensions due to disability. In the recent years, there is a growing proportion of new disability cases due to mental disorders (increased from 15.3 in 1995 to 35.3% in 2019) and neoplasms (from 9.5 to 14%, correspondingly). The proportion of disability pensions due to heart diseases declined between 1995 and 2019 but nevertheless occupies the third position among all the causes with 13.2% of the total number of disability pensions in 2019 (German Federal Pension Fund, 2020). Regarding the regional differences, the proportions of these diseases slightly vary between East and West. For instance, the shares of neoplasms and heart problems are bigger in the East than in the West (16% vs.13% for neoplasms and 15% vs.13% for heart diseases), while the mental disorders have larger proportion in the West (36% vs. 32% in 2019) (German Federal Pension Fund, 2020).

While some efforts have been made to evaluate socioeconomic differences among people receiving DP based on the diagnoses associated with pensioners’ disabilities, the number of demographic studies that have specifically examined the disabled population in Germany remains small. One of the examples is the work of Bäcker (2012) who provided an intensive summary on the structure of trends in and actual problems with the disability pension in Germany over the period 1993–2011. The author highlighted the difficulties in analyzing the disability trends over time when demographic changes are not taken into account. Cohort analysis is necessary to account for the aging of the baby boomers, which causes the number of DP recipients to increase, while the number of those starting old-age pensions decreases (Bäcker, 2012).

### Disability Pension in Germany

In its current form, DP (*Erwerbsminderungsrente*) was introduced on January 1, 2001. Two types of pensions are available: full and partial DP. To become eligible to receive this pension, an individual must have a long-term (at least 6 months) inability to work at least 6 h a day in the case of partial disability, or at least 3 h in the case of full disability. The assessment is “record-based”; i.e., it is based on medical records provided by a doctor. Because of the very strict evaluation criteria, only about 65% of all applications are accepted (Riphahn, 1997). To qualify to receive this pension, the individual must have completed the so-called “waiting time”, or at least 5 years of employment with paid contributions, 3 years of which were during the last 5 years. More information on the DP can be found elsewhere (Bäcker, 2012; Bundesministerium für Arbeit und Soziales, 2014; Hagen & Himmelreicher, 2014; Hanel, 2010; Kaldybajewa & Kruse, 2012). Selected official statistics as well as description of DP before 2001 are provided in the *Online Supplementary Material (Description and Table A1)*.

## Aim and Objectives

While the body of the literature exploring the possible reasons for the East–West mortality gap is large, the question of why the gap in life expectancy among men has been so persistent has yet to be answered. We hypothesize that East German men are less healthy than their Western counterparts, independent of their socioeconomic status (SES).

Given the strict eligibility criteria and the stringent procedures for obtaining DP, we assume that the obtaining DP is an objective measure of an individual’s health status (compared to, for example, self-perceived health or self-evaluated activities of daily living). Keeping in mind that non-communicable diseases define both mortality and disability, we approximate severe health limitations by receiving DP, which in turn is related to mortality (as it is already discussed in Sect. 1.2). As receiving DP indicates an individual´s severe health conditions (which in turn, largerly influence the transitioning to DP), we assume that this transitioning is independent of SES, and expect that the East–West mortality gap attenuates when the role of DP is accounted for. More specifically, this study has three objectives: (1) to analyze the level of mortality and the mortality trends among the male population receiving DP in East and West Germany; (2) to evaluate the impact of the compositional changes in DP recipients (i.e., the effect of changes in the prevalence in DP) on the evolution of the East–West mortality gap over time; and (3) to determine whether the risks of transitioning to collecting DP differ between East and West Germany, and whether these differences are related to SES.

## Data and Methods

Our analysis covers the period 1995–2013 and refers to the male population aged 30–59 years. It is based upon three datasets: (i) data from German Pension Fund (DRV or Deutsche Rentenversicherung Bund), (ii) German Socioeconomic Panel (GSOEP) data, and (iii) data from the Human Mortality Database (HMD).

To estimate the absolute size and mortality of the population receiving DP (DP population), we rely on the Scientific-Use files (SUF) of the DRV data for the years 1995–2013 (SUFRTBNjjXVSTDemo and SUFRTWFjjXVSTDemo), which are the large representative samples of anonymized individual records. The dataset consists of two parts: a population sample (*Bestand*) containing 1% of all DRV records (i.e., pension payments at the end the reporting year), and a death events sample (*Wegfall*) accounting for 10% of all death counts that occurred during the reporting year.^1^ Pension records and death counts are separate datasets that cannot be linked individually (Kibele, 2012). Over the period 1995–2013, the *Bestand* sample contains an average of about 180,000 anonymized pension records per year, of which 17,500 records refer to pensioners receiving disability pensions. The corresponding figures for the *Wegfall* sample are about 72,000 and 5,000 death counts, respectively. The share of people aged 30 years and above covered by the DRV data constitutes about 80–90% of total population (Scholz, 2011).

These large representative samples allow us to estimate first the size of the male DP population by 5-year age group and region (East/West) at the end of each reporting year. To account for the problem with different sample sizes, we applied the adjusting weighting procedure. Since the *Bestand* sample contains 1% of all DRV records, the weighting factor of 100 is applied to estimate the number of individuals receiving DP in the total population of Germany. The mid-year DP population is estimated as the average of the two successive years. Similarly, we estimate the absolute number of DP deaths by age and region, while weighting each record in the *Wegfall* sample by a factor of 10.^2^ We then use these results and HMD data to estimate the size of the male non-DP population and the corresponding number of deaths as the difference between the total male population (HMD) and the DP population. For both subgroups, we compute age-specific death rates. To account for differences in population age structures, we compute standardized deaths rates (SDR) using the German national population for the year 2005 as the standard population. Because the number of men with disabilities of younger working ages is relatively small, we used a 3-year moving average in estimating the SDRs to stabilize the estimates calculated from small numbers. To decompose the difference in the SDRs into mortality and composition components, we use the stepwise replacement algorithm. The detailed explanation of the method is provided in Andreev et al. (2002) and Andreev and Shkolnikov (2012). This approach can be used to break down the difference between two rates into the direct component (change in mortality) and the compositional component (change in health status/disability). To estimate the prevalence in DP and the relative risk of transitioning to DP, we rely on GSOEP data. The GSOEP is an ongoing survey of individuals, families, and households that began in 1984 with about 12,000 respondents aged 16 or older living in West Germany. After German reunification, the survey was extended to cover all of Germany. Information about survey is provided elsewhere (Haisken-DeNew & Frick, 2005; Kroh, 2014; Wagner et al., 2007).

The high-income sub-sample (G), the innovation sample (I), the samples on different birth cohorts and family types (L1–L3), and the migration sample (M1) are excluded from the analysis, because different selection procedures were applied for these samples. The final sample used for the estimates consists of 11,104 men with almost 72,000 observations. To adjust for the disproportionate sampling of subgroups and for non-responses, we applied a weighting procedure described in Kroll and Lampert (2009). Selected summary statistics is provided in Table A2 in the supplementary material.

To estimate the relative risk of transitioning to DP among men aged 30–59 years, we applied Cox proportional hazard models. The dependent variable — i.e., receiving DP for the first time — is a dichotomous measure indicating whether an individual received pension at any time after the first observation. Only those who participated in the survey at least two times are considered. Cases are censored upon leaving the survey if no DP were previously reported. The GSOEP questionnaire does not contain a direct question about whether the respondent receives DP. However, DP recipients can be identified indirectly by looking at those who were receiving pension before the age of 60 as this is the only way to retire before this age (Krause et al., 2013). Beginning from age 60 and until the official retirement age, several other options of early retirements become available (e.g., by long-term insured, by those who use the part-time retirement option or receive old age unemployment pension). A detailed description is available in Börsch-Supan and Jürges (2011).

The proportionality assumption has been verified by exploring the Schoenfeld residuals and by incorporating into the models the interactions of the covariates with function of time. As no statistically significant interactions were found (results not shown here), we can confirm that there is no violation of the proportionality assumption.

To assess the effect of different factors on the risk of transitioning to DP, the following covariates are used:

*Age* is a continuous variable that represents the individual’s age at the time of the interview.

*Region* is the categorical variable that separates men living in East and West Germany.

*Self-perceived health (SPH)* represents the time-varying covariate stratified into five categories: very good, good, satisfactory, bad, and very bad health. It is constructed based on the current health status variable (ple0008).

To evaluate whether the risk of transitioning to DP depends on an individual’s socioeconomic status (SES), the *highest level of education* is used and categorized into three groups based on the International Standard Classification of Education (ISCED-1997): lower secondary education (“low”), upper secondary education (“middle”), and tertiary education (“high”) (UNESCO, 2006). The cases with no available information on education are recoded as missing cases.

*Long-term unemployment benefits (ALG II)* were introduced in Germany as a part of the “Harz reforms” in the labor market to address high unemployment after reunification. We use ALGII receipt as an approximation of the individual’s SES in the context of reunification. *Receiving ALGII* is constructed as a dummy variable with two categories: (i) receiving and (ii) not receiving these benefits.

Two separate measures are introduced alternatively into the model to evaluate the potential effect of migration on the incidence of DP: *born in Germany* or immigrated before 1949 (yes or no) and *migration background* (no; first generation, or those with direct migration experience; and second generation, or those with indirect migration experience, i.e., whose father or mother had migration background).

The *interactions between region* and *SPH,* and *region* and *ALGII* are included as separate covariates to assess whether the risk of transitioning to DP by health status/receiving ALGII differs between the two regions.

## Results

### General Mortality Trends

**Fig. 2:**
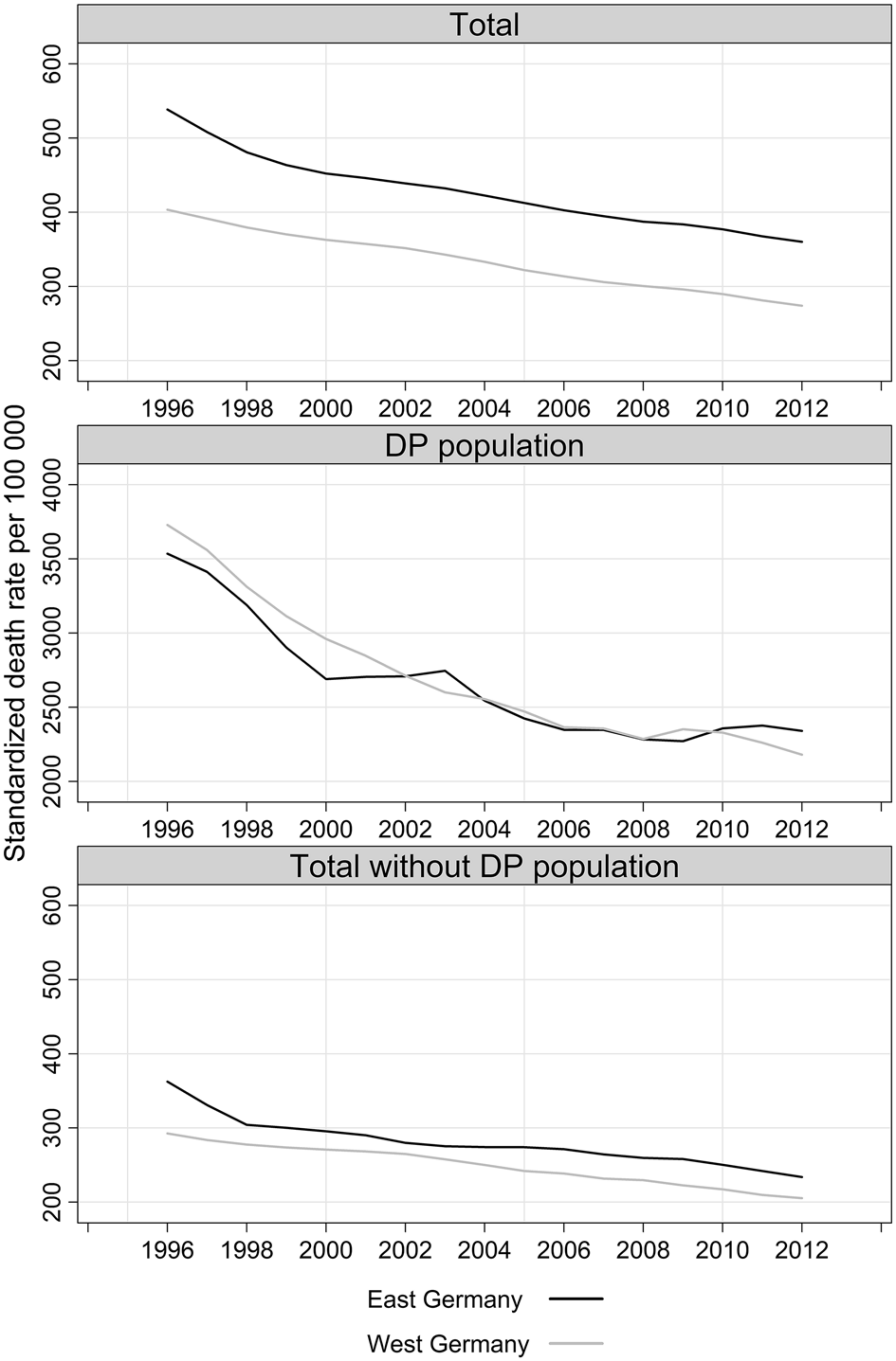
SDR for men aged 30–59 in East and West Germany before and after exclusion of the DP population, 1996–2012 (per 100,000) *3-year moving average * Source: own estimates from HMD and DRV data

In 1996, the 3-year moving average of the standardized death rate for people aged 30–59 was 539 deaths per 100,000 in East Germany, or 135 deaths per 100,000 more than in West Germany (Fig. 2). This East–West difference declined to 93 deaths per 100,000 in 1999 and has since then been almost unchanged (between 86 and 91 deaths per 100,000).

After men receiving DP were excluded from the total male population, the mortality gap between East and West became smaller. For instance, when only men without DP were considered for 2012, the rates declined to 205 per 100,000 in the West and 234 per 100,000 in the East, with the difference decreasing to about 28 deaths per 100,000 (Fig. 2).

Mortality among men with DP did not differ between the two regions. While in the beginning of the analyzed period, the SDRs were slightly higher among men in West than in East Germany; between 2002 and 2009, the regional trends were crossing each other. Recently, the SDRs among men with DP became persistently higher in the East.

The results from DRV data demonstrated that while those receiving DP made up only a small fraction in the male population, they accounted for more than one-third of deaths among men aged 30–59 (*Fig. F1* *in the Online Supplementary Material*). In terms of regional variation, the proportion of men receiving DP in the total male population was higher in the East (around 5%) than in the West (around 3%). This was also the case for the proportion of deaths among male DP recipients in the total number of deaths: In the East, their share varied between 36 and 43% in total deaths; while in the West, it ranged between 26 and 33% in total deaths.

The mortality gap in the non-DP populations in terms of SDRs was 30–40 deaths per 100,000 population, or two to three times lower than the levels in the total population. At the same time, the East–West mortality gap among the non-DP population was negligible.

### Prevalence of Receiving DP

**Fig. 3:**
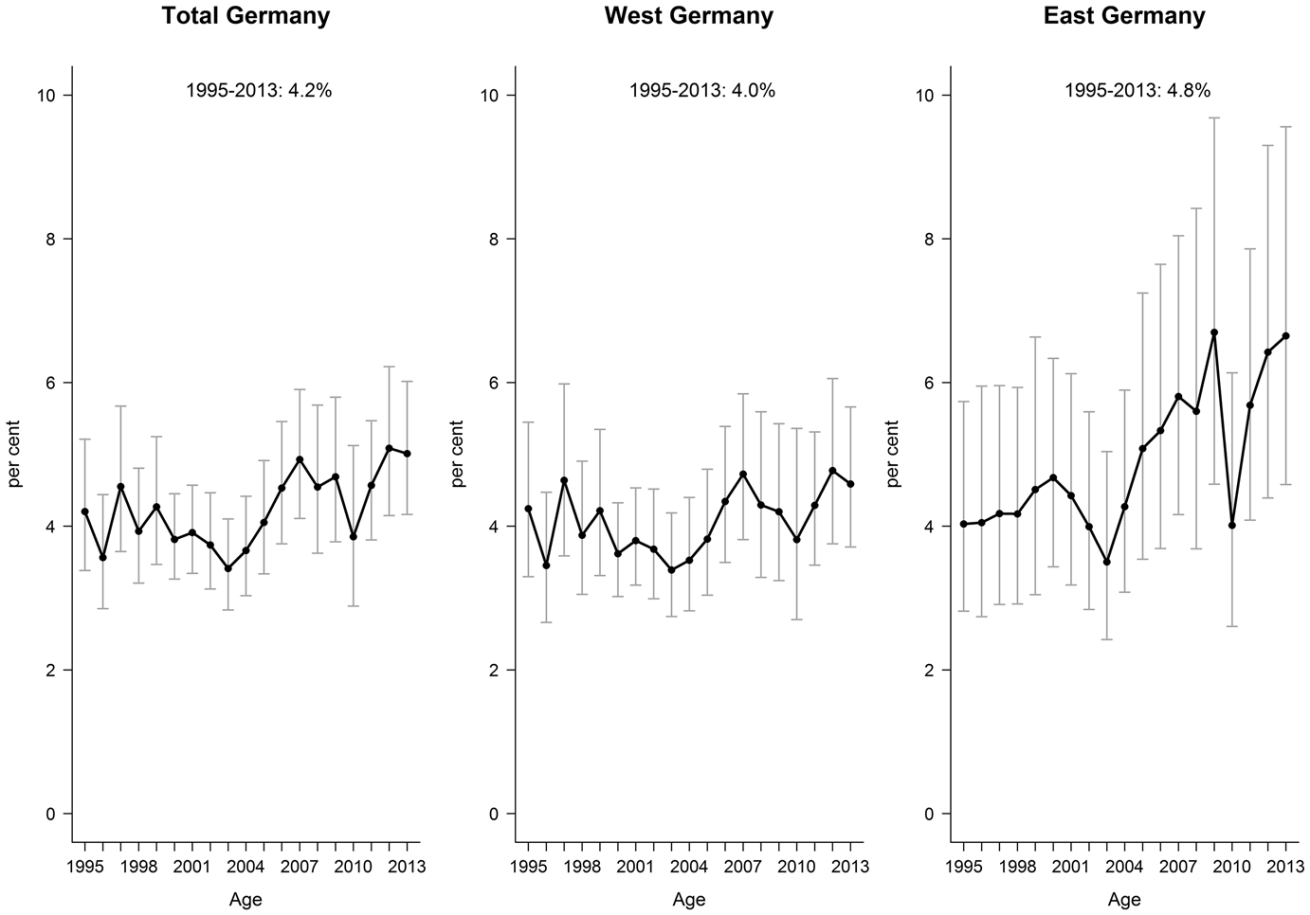
Proportion of men aged 30–59 receiving DP in Germany and by regions, 1995–2013 (per cent) Source: Own estimates from GSOEP data

According to the GSOEP data, over the whole analyzed period, the prevalence of receiving DP constituted 4.2% for Germany, 4% for the western part and 4.8% for the eastern part. During 1995–2013, the proportion of men receiving DP living in both parts of Germany varied between 3 and 7%. However, these data also confirmed the higher prevalence of DP recipients among men in the East (Fig. 3). There are some fluctuations in the trends for both East and West of Germany, which might be related to the effect of various policies and programs introduced for pension system in general, for disability pension system in particular and for the labor market system (more on this can be found in the discussion part).

### Decomposition Analysis

**Fig. 4:**
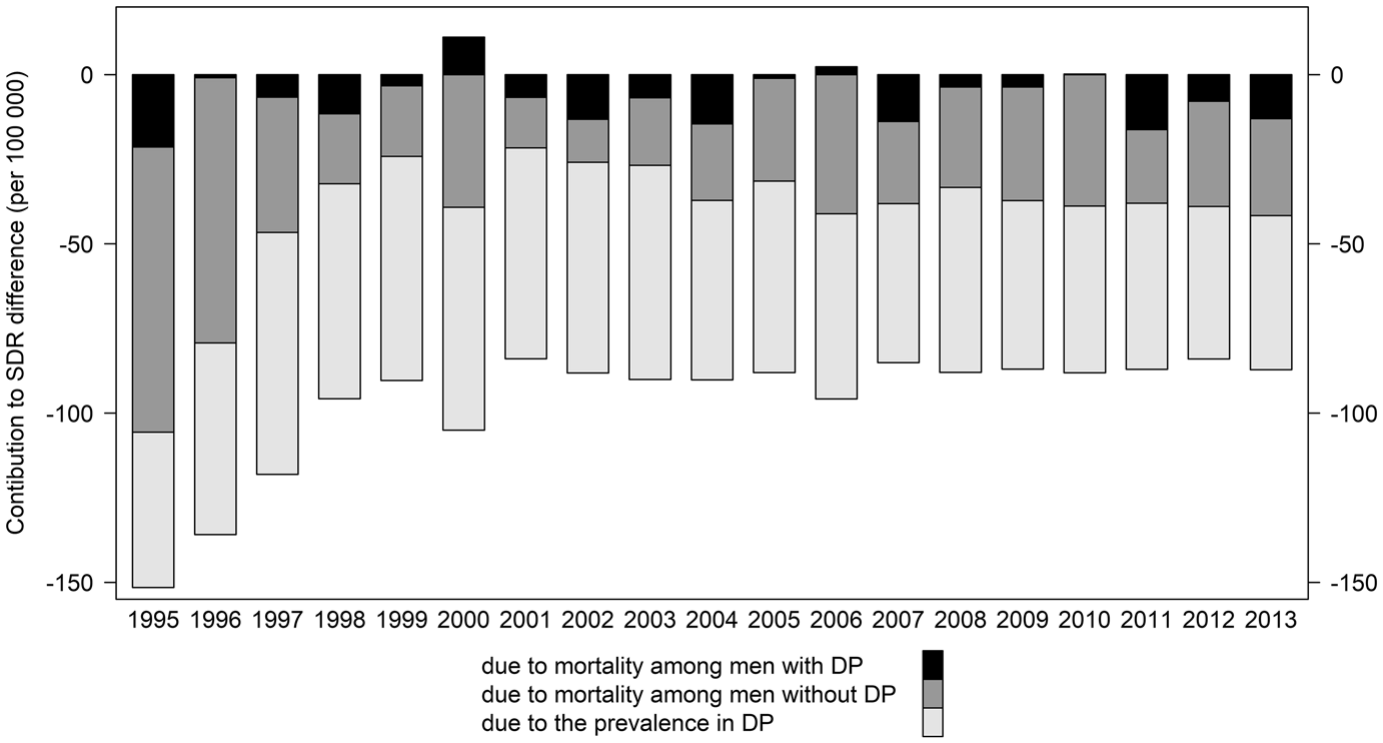
Decomposition of the East–West difference in SDRs by effects of mortality levels and population composition among men aged 30–59 (per 100,000); 1995–2013 Source: own estimates from HMD and DRV data

The results of the decomposition analysis are presented in Fig. 4 and Table 1. Negative values indicate the advantage of West over East. Each bar represents the total SDR difference between two regions. The contributions of each component are depicted by the corresponding colors.

**Table 1:** Decomposition of the East–West difference in SDRs by effects of mortality levels and population composition among men aged 30–59 (per 100,000); 1995–2013 *Source: own estimates from HMD and DRV data*

Year	Differences in SDRs due to:			Total difference
	Mortality among men without DP	Mortality among men with DP	Prevalence in receiving DP	
1995	− 84.3	− 21.4	− 45.9	− 151.6
1996	− 78.3	− 0.9	− 56.6	− 135.8
1997	− 39.9	− 6.7	− 71.5	− 118.1
1998	− 20.7	− 11.6	− 63.5	− 95.8
1999	− 20.8	− 3.3	− 66.2	− 90.3
2000	− 39.2	11.1	− 65.8	− 93.9
2001	− 14.9	− 6.7	− 62.3	− 83.9
2002	− 12.7	− 13.2	− 62.2	− 88.1
2003	− 20.0	− 6.9	− 63.2	− 90.1
2004	− 22.6	− 14.6	− 53.0	− 90.2
2005	− 30.4	− 1.1	− 56.6	− 88.1
2006	− 41.1	2.3	− 54.7	− 93.5
2007	− 24.3	− 13.9	− 46.9	− 85.1
2008	− 29.6	− 3.7	− 54.7	− 88.0
2009	− 33.6	− 3.7	− 49.8	− 87.1
2010	− 38.9	0.2	− 49.2	− 87.9
2011	− 21.7	− 16.3	− 49.1	− 87.1
2012	− 31.1	− 7.8	− 45.0	− 83.9
2013	− 28.6	− 13.0	− 45.5	− 87.1

The results of the decomposition analysis showed that the main driving factor contributing to the differences in the SDRs between the two regions was the compositional effect, followed by the differences in mortality among people not receiving DP (Fig. 4). For all years combined, the higher proportion of those receiving DP in the East explained more than half (about 60%) of the East’s disadvantage. About one-third (34%) of the disadvantage was explained by differences in mortality levels among men without DP, while the contribution of the mortality differences among DP recipients was minor (about 7%).

In 1995, the total difference in SDRs between two regions was 151.6 deaths per 100,000, of which 84.3 deaths (or 55.6% of total difference) were due to higher mortality in the East among men not receiving DP. There were 21.4 deaths (or 14.1%) because of higher mortality in the East among those receiving DP, and 45.9 deaths (or 30.3%) because of the higher proportion of people with DP in the East. In 2013, the corresponding differences constitute 28.6 deaths (32.8%), 13 deaths (14.9%) and 45.5 deaths (52.2%); all in the favor of the West.

Only in 1995–1996, the differences between men who did not receive DP contributed most to the difference in mortality rates. Since 1997, differences in the prevalence of receiving DP have become the main factor explaining more than half of the regional mortality difference. Mortality differentials for men receiving DP varied over the analyzed period, which, in addition to data issues, may be related to the introduction of various policies and reforms of the pension system, as well as the reforms and changes in the labor market (e.g., changes introduced in 2001; introduction of long-term unemployment benefits, etc.).

### Relative Risk of Transitioning to DP

There was no difference in the hazard of transitioning to DP between the two regions (HR = 1.0, *p* value = 0.97) when controlling for *age* (Model 1 in Table 2). Introducing *SPH* and *education* (Models 2 and 3) did not change the result. When *receiving ALGII* was considered, the direction changes such that people in the West had a higher hazard of transitioning to DP than people in the East; but the results were not statistically significant (Models 4–6).

**Table 2:** Cox hazard ratios (HR) of transitioning to DP among men aged 30–59; Germany, 1995–2013 *Source: own estimates based on the GSOEP data*

	Model 1		Model 2		Model 3		Model 4		Model 5		Model 6	
	HR	CIs	HR	CIs	HR	CIs	HR	CIs	HR	CIs	HR	CIs
Age	1.15***	1.13;1.17	1.12***	1.10;1.14	1.11***	1.09;1.14	1.12***	1.10;1.14	1.12***	1.10;1.14	1.12***	1.09;1.14
Region (Ref.: West)												
East	1.00	0.77;1.32	1.02	0.78;1.33	1.05	0.81;1.38	0.99	0.76;1.28	0.91	0.70;1.19	0.91	0.70;1.19
SPH (Ref.: Very good)												
Good			1.31	0.67;2.54	1.29	0.66;2.50	1.30	0.67;2.53	1.29	0.66;2.51	1.30	0.66;2.51
Satisfactory			2.28***	1.19;4.35	2.20**	1.15;4.22	2.18**	1.14;4.19	2.15**	1.12;4.13	2.15**	1.12;4.13
Poor			6.80***	3.57;12.93	6.51***	3.41;12.44	6.24***	3.27;11.92	6.19***	3.24;11.81	6.18***	3.24;11.81
Very poor			19.85***	10.22;38.54	18.79***	9.60;36.75	17.20***	8.82;33.57	17.12***	8.79;33.35	17.12***	8.79;33.35
Highest level of education (Ref.: Low)												
Middle					0.95	0.69;1.31	0.95	0.69;1.31	0.87	0.62;1.22	0.87	0.62;1.22
High					0.76	0.52;1.13	0.82	0.56;1.20	0.75	0.51;1.11	0.75	0.51;1.11
Receiving ALGII (Ref.: No)												
Yes							2.05***	1.54;2.73	2.17***	1.63;2.89	2.17***	1.63;2.90
Born in Germany (Ref.: Yes)												
No									0.68**	0.49;0.96		
Migration background (Ref.: No)												
1st generation											0.69**	0.49;0.96
2nd generation											1.04	0.64;1.68
Wald chi^2^	244.12		726.36		3156.74		5711.14		4243.30		5618.05	
Prob > chi^2^	0.000		0.000		0.000		0.000		0.000		0.000	

	Model 7	Model 8		
	HR	CIs	HR	CIs
Age	1.12***	1.10;1.14	1.12***	1.10;1.14
Region (Ref.: West)				
East	1.13	0.31;4.17	0.89	0.66;1.19
SPH (Ref.: Very good)				
Good	1.25	0.59;2.67	1.29	0.66;2.51
Satisfactory	2.24**	1.08;4.66	2.16**	1.12;4.15
Bad	6.52***	3.15;13.51	6.20***	3.25;11.85
Very bad	17.62***	8.31;37.36	17.16***	8.81;33.41
Highest level of education (Ref.: Low)				
Middle	0.87	0.62;1.22	0.87	0.62;1.22
High	0.75	0.51;1.12	0.76	0.51;1.12
Receiving ALGII (Ref.: No)				
Yes	2.17***	1.63;2.89	2.11***	1.12;2.94
Born in Germany (Ref.: Yes)				
No	0.68**	0.49;0.96	0.69**	0.49;0.96
Interaction SPH*Region (Ref.: Very good * East)				
Good * East	1.06	0.25;4.53		
Satisfactory * East	0.77	0.18;3.23		
Bad * East	0.70	0.18;2.75		
Very bad * East	0.81	0.20;3.23		
Interaction ALGII*Region **(**Ref.: Yes, West)				
Yes, East			1.12	0.60;2.10
Wald chi^2^ (degree of freedom)	806.27		5928.36	
Prob > chi^2^	0.000		0.000	

CIs: 95% confidence intervals; SPH: self-perceived health; ALGII: long-term unemployment benefits

***p ≤ 0, 01; **p ≤ 0,05; * *p* ≤ 0,1

As expected, we found that the relative risk to transit to DP increased with *age* and was strongly associated with *SPH*. For each additional year of life, the hazard rate increased by 11–15% (*p* value < 0.01). Compared to being in very good health, being in poor health increased the hazard six to sevenfold, while being in very poor health increased the hazard as much as 17–20-fold (Models 2–8).

The results showed that *education* had no effect once health was controlled for (Models 3–8), but that *ALG II* recipients had twice the hazard of transitioning to DP (HR = 2.1–2.2, *p* value < 0.01; Models 4–8). There was a clear effect of *migration background*: men born outside of Germany had a roughly 30% lower relative risk receiving DP (HR = 0.7, *p* value = 0.03; Model 5). Men with a direct migration background had the lowest hazard of transitioning to DP relative to that of non-migrants (HR = 0.7, *p* value = 0.03; Model 6). No statistically significant effect was found for the second-generation migrants (HR = 1.04, *p* value = 0.89).

The *interaction between SPH* and *region* was found to be statistically insignificant (Model 7). This was also true for the *interaction between region* and *receiving ALGII* (Model 8).

## Discussion

### Main Findings

This study explored the impact of mortality among men receiving pension due to disability on the evolution of the East–West mortality gap in order to test the hypothesis that the persistent gap in life expectancy is partly attributable to the generally worse health of East German men. To our knowledge, this is the first study to consider the contribution of the group with severe health limitations to overall mortality differences.

We used receiving DP as an indicator of severe disability. We chose this indicator because it is one of the most objective ways to approximate an individual’s health status (namely severe health problems). Since eligibility for this pension is determined using strong criteria based on an individual medical assessment, receiving DP is a much more objective health indicator than, for instance, self-perceived health. Another reason for using DP in mortality analysis is that mortality risk is known to be higher among disabled individuals compared with the general population. Moreover, both mortality and disability are caused mainly by the non-communicable diseases (e.g., neoplasma and heart problems). Our results revealed that although the share of the DP population in the whole population was small, it accounted more than one-third of the deaths among adult men of working ages. During the analyzed period, death rates were much lower in the West Germany when all men were considered. The difference in mortality between the two regions declined after men receiving DP were excluded from the estimates; yet disparities remained in favor of the West. When only men receiving DP were considered, there existed no clear and big difference between the two regions.

The previous intensive literature dealing with the analysis of regional mortality differences linked the lower mortality in the West Germany to the better individual’s socioeconomic status in the region and to the differences in the individual’s behavior (smoking and drinking habits, diet or stress). Factors such as income, education or occupation were found to play a contributing role in defining the regional differences in mortality (Grigoriev et al., 2019). Empirical data, however, do not always provide expected and consistent results about the impact of SES on mortality or disability and attention should be paid to the choice of the indicators used for approximating SES (e.g., income or education) and health (e.g., disability or self-perceived health). The impact of income-related differences on mortality might give different results as compared to the effect of education on disability. According to the GSOEP data, during 1995–2013, the proportion of less-educated men was considerably lower in the East Germany (varied between 2 and 5%) as in the West (varied between 8 and 12%), yet a larger share of men receiving DP was found in the East (prevalence in receiving DP by education is available in Figure F2 in the *supplementary material*).

As the decomposition analysis showed, the difference in the prevalence in receiving DP between the East and the West was the main factor to contribute to the regional mortality gap which explained more than 60% of the disadvantage of the East over the whole analyzed period. It could be an artifact caused, for instance, by the differences in the assessment or application procedures to obtain DP in the two regions. Potential differences in DP attainment between the two regions could be, for example, when a person is unable to perform a current job due to health limitations but has a potential to perform the tasks of a similar skill; or when a person is unable to find other work due to limited job opportunities in a particular region. However, the influence of such factors is difficult to capture due to the lack of necessary data. Overall, such an artifact was not supported by the regression results: No statistically significant effect of the region on the hazards of transitioning to DP was found (neither in non-adjusted nor in adjusted models). Also, it should not be the case due to the objective nature of the measure used in this work to approximate health: It is very difficult to obtain DP unless an individual has serious health problems. Considering all, the compositional difference seemed to be the real effect rather than the artifact.

The less favorable composition among the East German population in terms of important structural characteristics was also confirmed by the previous research. For instance, the differences in age, employment status, insurance status and nationality led to a higher mortality rate in the East, especially for men (Scholz, 2011). The East–West difference in mortality among men not receiving DP also contributed to the regional mortality gap but to a lesser extent than the compositional effect. Among the factors that were previously shown to explain the persisting East–West gap in mortality among economically active men was higher individual’s income in the West (Grigoriev et al., 2019). In addition, post-reunification stress in the East might have also played a role (Diehl, 2008). The contribution of the differences in mortality among men receiving DP to the regional disparities constituted over the whole analyzed period only about 7%. The analysis of the impact of SES on this difference did not provide any conclusive results. Since the proportion of better-educated men was higher in the East, we suppose that the remaining East–West difference is not related to the SES but rather to the general mortality disadvantage of men in the East in all socioeconomic groups.

Regarding the relative risk of transitioning to DP, the results did not show any clear effect of region, which suggests that the assessment procedure for obtaining DP did not differ between the East and the West. The finding that a higher proportion of men had DP in the East suggested that adult West German men were healthier than their counterparts living in the East.

Given that health has been shown to be highly related to an individual’s socioeconomic status (Mackenbach & Kunst, 1997; Mackenbach, 2006; Culter and Lleras-Muney 2006; Mirowsky & Ross, 2003; RKI 2016), it can be assumed that a similar relationship existed for collecting disability pension. However, according to our regression results, the relative risk of transitioning to DP purely depended on health. Education, which approximated the individual’s SES, showed no statistically significant results when health was accounted for, while there was a very strong relationship between self-perceived health and the relative risk of transitioning to DP, with the least healthy people having the highest hazard of transitioning to DP. The association between education and receiving DP when no other factors are considered (results not shown here) holds only for those in the higher socioeconomic group: These men have lower relative risk of receiving DP. According to the GSOEP data (Table A2 in the supplementary material), the share of people with low education in the East is small (2%) as compared to the Western part (10.5%). Considering this and the fact that prevalence rate of receiving DP is higher in the East supports our conclusion that receiving DP is not SES- but health-dependent. Several income-related variables (per capita household income or individual income) were incorporated into the models (results not shown here), but the results showed no effect on the hazard of transitioning to DP. For those with average SES, the results might suggest that once they become disabled (or in need of care) other factors than socioeconomic status may start playing a role (Grigoriev & Doblhammer, 2019). The choice of the variable used to approximate the SES was also important, as different results were obtained when different measures were applied. Unlike education, when previous wages were used as the SES proxy, they were found to be strong predictors on the transition to retirement due to disability (Hanel, 2010; Riphahn, 1999).

Receiving long-term unemployment benefits (ALGII) was shown to have a positive and strong association with the relative risk of receiving DP among adult men. The relationship between DP and ALG II benefits merits particular attention in this context. The slight increase in the share of men receiving DP starting from around 2005 could be explained by the introduction of the *Hartz IV* reform, which combined the previously available unemployment assistance (*Arbeitslosengeld*) and social assistance (*Sozialhilfe*) benefits into the new long-term unemployment benefits II program (*Arbeitslosengeld II*) (Königs, 2014; Mika & Lange, 2014; Ochel, 2005). Before 2005, only those who were receiving short-term unemployment benefits could apply for DP. In the 2005–2010 period, those receiving ALG II benefits could also apply for DP because they were contributing to the pension fund, and their time in unemployment could be used to meet the waiting time criteria. Since 2011, pension contributions are no longer paid for individuals receiving ALG II benefits, which means that they are no longer included in the pension scheme (Bäcker, 2012; Mika & Lange, 2014). Mika and Lange (2014) showed that of the men who started receiving DP in 2010, 28.4% did so after receiving ALG II benefits for at least 36 months in the 2005–2010 period. The proportion of these individuals with very low entitlement levels was large enough to influence the average level of DP (Mika & Lange, 2014). To evaluate whether there were any regional disparities in the association between receiving ALGII benefits and the transition to DP, the interaction between ALGII and region was introduced into the regression analysis. The results were not statistically significant, which suggests that the transition from receiving long-term unemployment benefits to receiving DP was similar in the two regions.

The results of the regression analysis were consistent with the *selective migration* explanation that benefits the West. Men born in Germany had a higher relative risk of transitioning to DP than men born outside of Germany. The differences were also pronounced when direct or indirect migration background was considered: i.e., men with a direct migration background had a lower relative risk of receiving DP than non-migrants.

Based on our results, we propose at least four explanations why men in the East have worse health, higher prevalence in DP and a general mortality disadvantage. First, unlike men living in West Germany, the younger cohorts of men living in East Germany were exposed to an abrupt increase in unemployment. According to some estimates, around one-third of all jobs in the East were eliminated during the first 2 years after reunification (Geiβler, 1996). Thus, the loss of employment and income after reunification, and the accompanying decline in socioeconomic status experienced by East German men earlier in life may have adversely affected their health later in life (Roelfs et al., 2011).

Second, selective migration might explain the disadvantaged position of East German men*.* Since reunification, younger and more skilled people have been migrating to the West from the East in order to take advantage of better employment opportunities. Those who are older, socially disadvantaged, or in need of care have tended to stay in the East. These trends have negatively affected the level of unemployment, the age and the sex balance of regional populations, and the regional social security systems (Razum et al., 2008). The East-to-West migration wave that started around 1997 coincided with a period of economic stagnation in the East and improvements in the job market in the West (Heiland, 2004). It has been suggested that high levels of regional income inequality have affected migration flows (Fuchs-Schündeld & Schündeln, 2009). There is also evidence that East–West migrants have been getting younger and more educated over time (Fuchs-Schündeld & Schündeln, 2009).

Third, in addition to East-to-West migration, the so-called “healthy migrant effect” among foreigners living in Germany, most of whom migrated to West rather than to East Germany (German Federal Statistical Office, 2022a, b), may have contributed to the advantage of the West. Guillot et al. (2018) observed that while migrants generally represent a highly selected population, relative migrant mortality can vary by age. Based on an analysis of data for France, the US, and the UK, the authors found evidence of excess mortality among younger migrants, a mortality advantage among migrants at adult ages, and a convergence in mortality levels between the older native population and migrants at older ages. A mortality advantage among the foreign population that is attributable to the health selection effect has also been reported for Germany (zur Nieden & Sommer, 2016)*.*

Last but not least, in addition to the healthy migrant effect, the “salmon bias hypothesis” is often cited to explain the better health observed among migrants and might, in turn, determine the population composition. According to this selective return migration hypothesis, unhealthy migrants or those whose health is deteriorating tend to return to their country of origin (Andersson & Drefahl, 2017; Lu & Qin, 2014). The return migration of non-Germans due to sickness has been documented for Germany (zur Nieden & Sommer, 2016).

### Strengths and Limitations

The DRV dataset we used in this study has several advantages, including its coverage, completeness, and long time series. Because the DRV includes all deaths, the data are not affected by attrition bias, as is usually the case for survey data. The major advantage of using this dataset is, however, the objective nature of the dependent variable (receiving DP). The GSOEP data are longitudinal, representative, and cover a wide range of demographic and socioeconomic variables.

This study has several limitations. First, although the DRV data cover the majority of the German population, some population groups, such as self-employed individuals and civil servants (*Beamte*), are excluded from the DRV records. Civil servants are a highly selected group of individuals with lower mortality compared to the general population (zur Nieden & Altis, 2017). Since the proportion of civil servants and self-employed is larger in the West than in the East, their inclusion in the analysis may exacerbate the disadvantage of the East.

Second, we were not able to extend our analysis to men aged 60 or older. This is because those who receive DP are transferred into the old-age pension system as soon as they reach the official retirement age. In Germany, there are several other options for early retirement, although it does not begin until after age 60 (Börsch-Supan & Jürges, 2011). The DRV data that were available to us did not include information about the transition from one type of pension to another. Therefore, we cannot distinguish what type of pension a person receives after reaching age 60. Similarly, the GSOEP data do not contain direct information on whether the respondent receives DP. They were identified indirectly by looking at those who received a pension before age 60.

DRV SUF files provide a very limited number of variables that could be used for the detailed analysis of the factors associated with severe disability. For example, the availability of information on the diagnosis that leads to disability would help to better understand how debilitating diseases contribute to regional mortality disparities.

In general, our analysis of the long-term trends in the disability pension system was hampered by the changes in how this pension was defined, and in the regulations determining who was eligible to claim it. During the period studied, several reforms were enacted that affected the pension system as a whole and the disability pension system in particular (Buchholz et al., 2013): These were aimed at extending the working lives of older people and closing various pathways to early retirement. For instance, since the reform of 2001, actuarial adjustments have been applied to the DP. As a result of this change, the DP have been reduced by 0.3% for each calendar month for recipients who retire before the age of 63 (Berkel & Börsch-Supan, 2004). These changes, as well as the ALGII benefits discussed above, may partly explain the variation in the prevalence of DP receipt over the analyzed period. However, since these reforms have been enforced uniformly in the two parts of Germany, and also, the fluctuations in the trends are observed in the same years in both regions, we believe that these changes should not influence our interpretation of the East–West mortality difference. Another limitation is that the number of migrants receiving the DP might underestimate the true burden of disability in this population due to the lack of 5 years of paid contribution to the pension system and/or lack of knowledge to apply for these benefits. Considering that the number and share of migrants is bigger in the West this might lead to some bias in the estimates. Unfortunately, we have no data to confirm this. On the other hand, higher unemployment after reunification has put men in the East in an unfavorable position. The long-term unemployed were eligible to apply only for a limited time, so they lacked the 5 years of paid contributions and the eligibility to apply for DP. In general, the higher proportion of disabled men in the East was confirmed by previous research (Hagen et al., 2010; Hagen & Himmeireicher, 2014).

## Supplementary Information

Below is the link to the electronic supplementary material.

Supplementary file1 (PDF 253 KB)

## Data Availability

Not applicable.

## Footnotes

1 The short description of the datasets can be found at: http://www.fdz-rv.de/FdzPortalWeb/dispcontent.do?id=main_fdz_forschung_querb&chmenu=ispvwNavEntriesByHierarchy48.

2 Similar weighting procedure was applied in previous research (Scholz and Schulz, 2007).
