# Supplementary material for: Does the Mortality of Individuals with Severe Disabilities Contribute to the Persistent East–West Mortality Gap Among German Men?
Source: Eur J Popul. 2022 Mar 1;38(2):247–71. doi: 10.1007/s10680-022-09609-4 (PMC9127014; doi:10.1007/s10680-022-09609-4)
Supplement: Supplementary file 1 — Supplementary file1 (PDF 253 KB) [file 10680_2022_9609_MOESM1_ESM.pdf]

Table A1. The number of new DP cases and the number of people receiving DP at the end of the year; total German population and men, 1995-2018 (thousands)

year	Number of new cases of DP				Number of people receiving DP at the end of the year			
	Total Germany	Germany, men	West, men	East, men	Total Germany	Germany, men	West, men	East, men
1995	294.0	184.3	149.5	34.8	1862.9	1105.1	876.3	228.8
1996	279.7	175.1	142.7	32.4	1918.2	1116.0	884.1	231.9
1997	264.2	165.6	132.5	33.1	1933.1	1115.0	878.5	236.5
1998	237.1	149.6	120.9	28.6	1936.1	1112.0	872.4	239.6
1999	218.2	137.4	111.3	26.1	1890.2	1083.6	848.5	235.2
2000	214.1	131.8	107.4	24.4	1894.0	1072.7	832.4	240.3
2001	200.6	119.9	96.9	23.0	1861.5	1043.8	805.7	238.1
2002	176.1	102.8	81.8	21.0	1809.1	1003.4	768.5	234.9
2003	174.4	100.5	79.7	20.8	1761.6	969.7	742.7	227.0
2004	169.5	96.6	76.2	20.4	1694.7	924.0	707.3	216.8
2005	164.0	91.4	72.1	19.3	1649.7	891.7	681.3	210.4
2006	159.7	89.2	70.8	18.4	1602.4	861.0	656.1	204.9
2007	161.5	89.4	70.4	19.0	1583.8	844.4	643.7	200.7
2008	162.8	88.0	69.4	18.6	1563.8	825.9	633.6	192.3
2009	173.0	92.3	71.9	20.5	1567.8	821.7	624.7	197.0
2010	182.7	96.7	75.9	20.7	1589.3	827.5	629.5	198.0
2011	180.2	94.6	74.9	19.7	1634.1	844.3	642.0	202.3
2012	178.8	92.2	73.1	19.0	1677.5	858.0	652.9	205.1
2013	176.7	90.1	71.3	18.8	1719.3	867.9	660.7	207.2
2014	170.8	86.6	68.8	17.8	1755.1	874.7	667.1	207.6
2015	174.3	87.4	69.6	17.9	1787.9	879.6	673.1	206.5
2016	174.0	86.1	68.7	17.5	1813.5	881.5	676.8	204.6
2017	165.6	82.1	65.0	17.0	1824.9	879.2	677.7	201.5
2018	168.0	81.5	64.4	17.1	1824.8	870.3	673.1	197.2

Sources: German Federal Pension Fund (2007, 2013, 2019). Rentenversicherung in Zeitreihen, Band 22.

Description of the disability pension (DP) in Germany before 2001

Before 2001, two types of DP were available to individuals with limited working abilities: (i) an occupational DP (*Rente wegen Berufsunfähigkeit*) for those whose ability to perform any work was less than half that of a healthy person in the same occupation; and (ii) a general DP (*Rente wegen Erwerbsunfähigkeit*) for those who were not healthy enough to perform their regular job duties, or for those who were receiving occupational DP, and were unable to find a part-time job within a year (Riphahn 1999). The level of benefits provided by a general DP was the same as that provided by a full old-age pension, whereas the level of benefits associated with an occupational DP was two-thirds that of a full old-age pension (Hanel 2010; Riphahn 1999). Of the DP applications that were approved, 80% were for general DP and 20% were for occupational DP (Riphahn 1999). Moreover, before 2001, general DP were provided to those individuals whose health status allowed them to work part-time, but who were unable to find a job within a 12-month period. After 2001, occupational DP were upgraded to general DP (Hagen and Himmelreicher 2014). Due to the enactment of new regulations for receiving DP, benefits for the partially disabled have declined since 2001.

Table A2. Summary statistics on the GSOEP data used in the Cox regression model: share of men by selected characteristics (%) and rates of the first transitioning to DP (per person-year)

Variables	Proportion by different characteristics, %		Rates of the first transitioning to DP (per person-year)	
	West	East	West	East
**Age groups**				
30-34	15.4	15.3	0.0004	0.0007
35-39	17.9	16.2	0.0016	0.0052
40-44	18.8	18.5	0.0036	0.0045
45-49	17.2	17.5	0.0079	0.0081
50-54	15.3	16.3	0.0093	0.0120
55-59	15.4	16.2	0.0297	0.0198
**SPH**				
Very good	8.7	6.1	0.0012	0.0020
Good	44.1	44.7	0.0027	0.0035
Satisfactory	32.6	34.8	0.0063	0.0067
Bad	11.8	11.3	0.0235	0.0190
Very bad	2.8	3.1	0.0784	0.0818
**Highest level of education**				
Low	10.5	2.0	0.0124	0.0186
Middle	54.3	61.9	0.0088	0.0077
High	35.2	36.1	0.0050	0.0081
**Receiving ALGII**				
No	96.7	92.2	0.0070	0.0074
Yes	3.3	7.8	0.0313	0.0155
**Born in Germany**				
Yes	84.5	98.7	0.0080	0.0080
No	15.5	1.3	0.0078	0.0137
**Migration background**				
No	78.1	93.1	0.0080	0.0081
1st generation	15.5	1.3	0.0078	0.0137
2nd generation	6.3	5.6	0.0077	0.0051

Source: Own estimates from GSOEP data

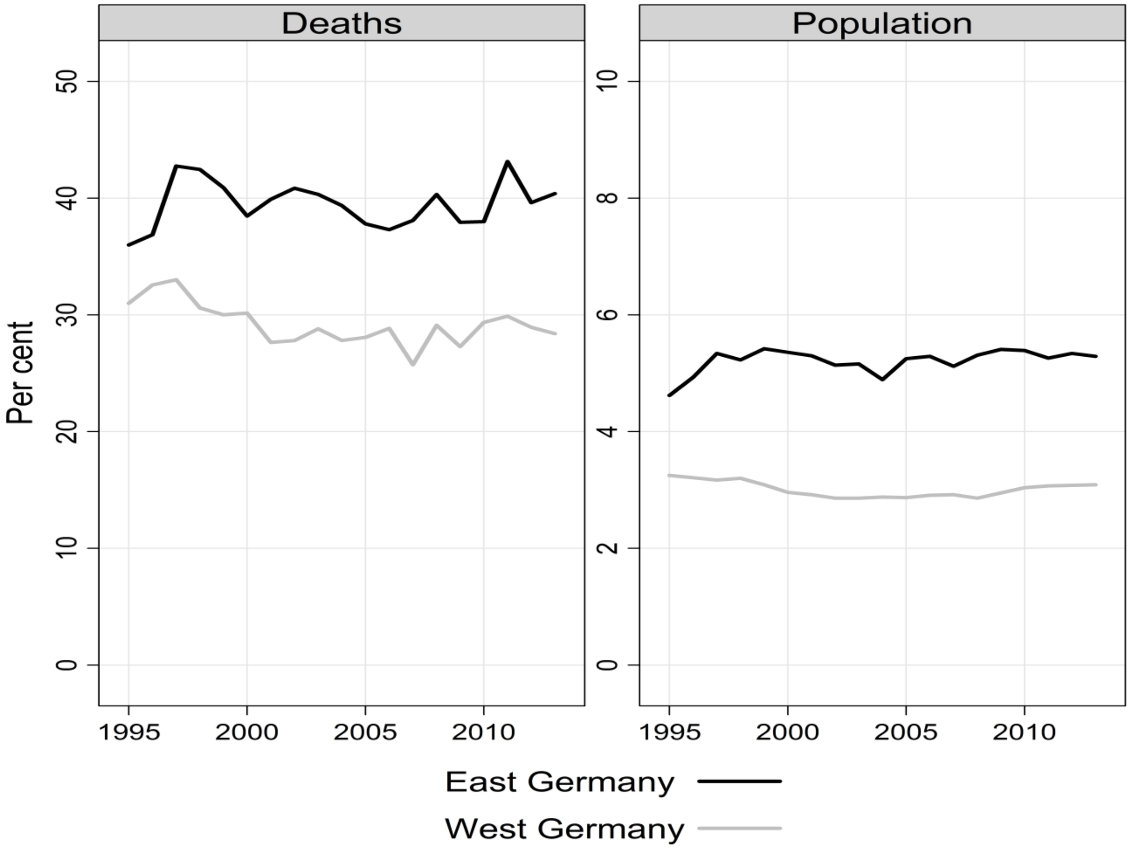

Fig. F1. Proportion of DP deaths in the total number of deaths and the DP population in the total population; men aged 30-59 in East and West Germany, 1995-2013 (percent)

Sources: Own estimates from HMD and DVR data

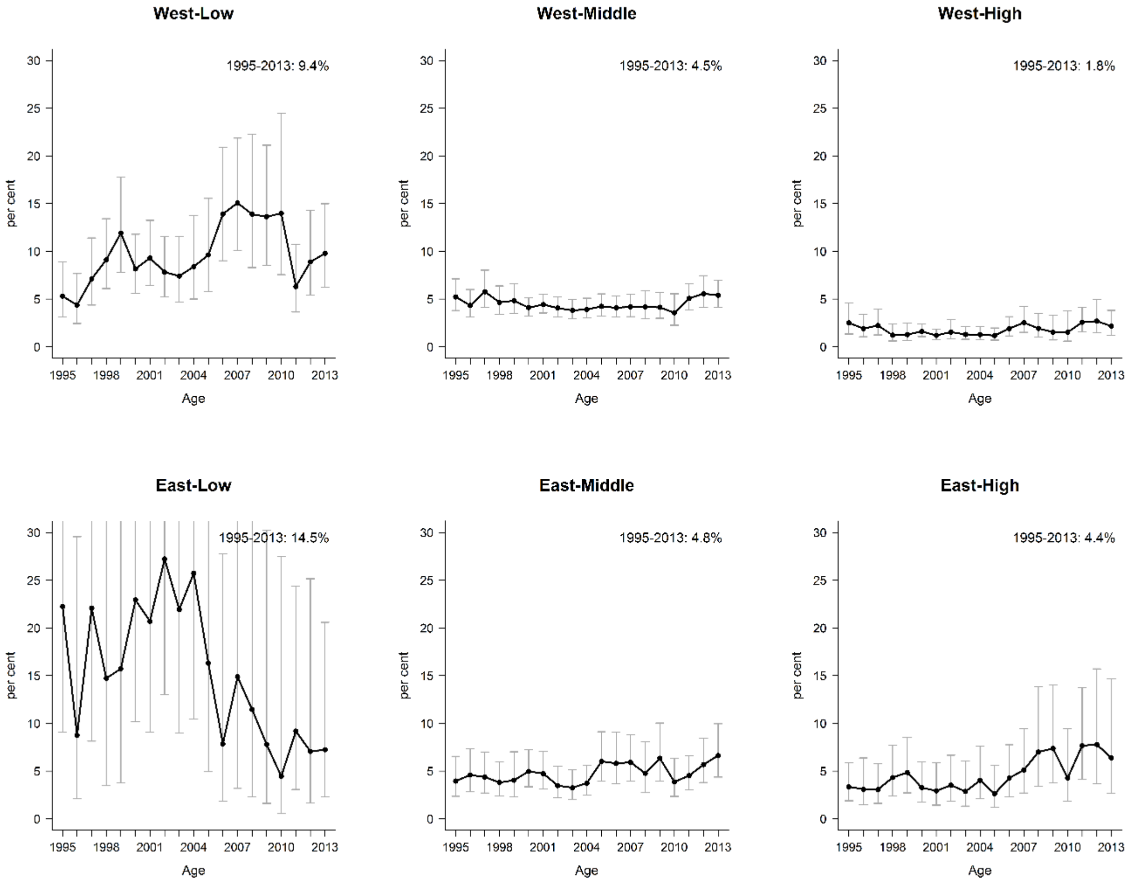

Fig. F2 Proportion of men aged 30-59 receiving and not receiving DP by educational categories in East and West Germany, 1995-2013 (percent)
Source: Own estimates from GSOEP data
